# Antibody-dependent fragmentation is a newly identified mechanism of cell killing *in vivo*

**DOI:** 10.1038/s41598-017-10420-z

**Published:** 2017-09-05

**Authors:** Pei Xiong Liew, Jung Hwan Kim, Woo-Yong Lee, Paul Kubes

**Affiliations:** Snyder institute of Chronic Diseases, University of Calgary, Calgary, Canada

## Abstract

The prevailing view is that therapeutic antibodies deplete cells through opsonization and subsequent phagocytosis, complement-dependent lysis or antibody-dependent cellular-cytotoxicity. We used high resolution *in vivo* imaging to identify a new antibody-dependent cell death pathway where Kupffer cells ripped large fragments off crawling antibody-coated iNKT cells. This antibody-dependent fragmentation process resulted in lethality and depletion of crawling iNKT cells in the liver sinusoids and lung capillaries. iNKT cell depletion was Fcy-receptor dependent and required iNKT cell crawling. Blood, spleen or joint iNKT cells that did not crawl were not depleted. The antibody required high glycosylation for sufficiently strong binding of the iNKT cells to the Fc Receptors on Kupffer cells. Using an acetaminophen overdose model, this approach functionally depleted hepatic iNKT cells and affected the severity of liver injury. This study reveals a new mechanism of antibody-dependent killing *in vivo* and raises implications for the design of new antibodies for cancer and auto-reactive immune cells.

## Introduction

Development of antibodies to eliminate target cells has become a hugely successful experimental and therapeutic approach. Despite their growing widespread use, with many antibodies moving into the clinical arena, the mechanism by which these antibodies function is still very poorly understood. It is however predicted that after the antibodies bind a target cell, they induce one of three forms of cell death: (1) antibody-dependent cell-mediated cytotoxicity (ADCC), (2) complement-dependent cytotoxicity (CDC) and (3) antibody-dependent phagocytosis^1, 2^. In ADCC-mediated cell death, the binding of cytotoxic cells (for example, NK cells) to antibody-opsonized tumor cells result in the release of vesicular contents such as perforin and granzymes which lyse and kill the tumor cells^3^. Although these antibodies can also activate complement to induce membrane disruption and cell death, this mechanism is not considered to be the dominant mechanism of killing^2, 4^. Opsonization of cells is also thought to induce phagocytosis. For example, intravital imaging work has shown that the anti-CD20 antibody (rituximab) which targets B cell lymphomas induces a phagocytic mechanism by Kupffer cells lining the liver sinusoids^5^. When opsonized B cells were injected into the mainstream of blood, they flowed towards intravascular Kupffer cells, were ensnared and phagocytosed resulting in cell death. Glycoengineered anti-CD20 antibodies improved the Kupffer cell-mediated phagocytosis of B cells^6^.

Recently, the use of therapeutic antibodies to target tumor cells has implicated trogocytosis, the process of ripping off or nibbling and internalizing small bits of the target cell membrane, instead of phagocytosis of whole cells^7^. This process has been shown to have varied results ranging from (1) removal of antibody from the target cell making it more pro-tumorigenic, (2) have no effect on the tumor or (3) through repeated trogocytosis of the tumor cell membrane, lead to increased tumor cell death and a decrease in tumor burden^7, 8^. Trogocytosis of target cells depends on a variety of factors including the type of target and effector cell, the degree of glycosylation which dictates the affinity for Fcγ receptors and behavior of cells^7–11^. Defining the pathway(s) of cell death has key implications for strategies in utilizing antibody-based therapies to treat different kinds of cancer.

Antibody directed immunotherapy is becoming an extremely promising strategy to target tumor cells in cancer but can also be used to target inappropriately activated immune cells in autoimmune disease. Indeed, while the anti-CD20 antibody is now regularly employed as a hematological cancer therapeutic and represents a breakthrough in the treatment of B cell malignancies^12–14^, these anti-CD20-specific antibodies, as well as CD52 specific alemtuzumab, Her2/neu-specific trastuzumab, EGRF-specific cetiuximab and anti-GD-2 antibodies are all under investigation in clinical trials to target depletion of both cancer and immune cells^10, 15–17^. Moreover, many new antibodies are now being developed to selectively deplete immune cells *in vivo*. For example, antibodies have been used to successfully deplete B cells^5^, natural killer cells^18–20^, dendritic cells^21^, neutrophils^22, 23^ and a variety of T cells including CD4^+^ T cells^24, 25^, CD8^+^ T cells^26, 27^, γδ T cells^28^, iNKT cells^29^ and Tregs^30^. Although it is presumed that the spleen and liver sequester these opsinized target cells via the same mechanism, many populations of cancer cells and immune cells might not flow through the mainstream of blood but instead transverse or crawl along the luminal surface of blood vessels. There is absolutely no information how a crawling cell might be eradicated. In addition, the size of target cells could limit the ability of the phagocytosing cell to engulf the target cell. Under these conditions, it is conceivable that a sufficiently large cell cannot be targeted and depleted by antibodies.

Immense effort has been made by biopharmaceutical companies to engineer third generation antibodies that can induce higher effector responses. Critical to all these events are Fc receptors. The constant fragment (Fc) of an antibody is necessary for interactions with immune cells and the efficacy of the antibody depends upon Fc modifications which occur during antibody production in the host. For example, absence of Fc glycosylation or certain modifications to amino acids in the Fc region dramatically reduces binding affinity^31–33^. Typically, third generation antibodies have increased Fc-linked glycosylation to improve ADCC although other mechanisms of killing may be enhanced concurrently^10, 34^. In fact, there are at least 40 therapeutic antibodies approved for human use and it is estimated that 30% of all new drugs will soon be antibodies but their mechanisms of action remain unclear^11, 31^. For example, the CC chemokine receptor 4 (CCR4)-targeting glyco-engineered antibody, mogamulizumab, has enhanced ADCC with no known explanation, but has recently been approved in Japan for use in patients with relapsed and refractory CCR4-positive adult T cell leukemia/lymphoma^35^. Similarly, although the complex interacting network between CXCR3 and its ligands or cellular expression have not been fully understood, companies have now patented the use of anti-CXCR3 antibodies in the treatment of cancer and autoimmune diseases^36, 37^. Regardless of mechanism, antibodies against specific cell surface proteins are a commonly used technique to deplete immune cell populations *in vivo*. Since most interrogations are done in cell culture systems devoid of blood flow and other important *in vivo* micro-environmental factors, the mechanisms of action of these antibodies remain equivocal.

In this study, we show a novel antibody-dependent cellular killing mechanism which is dependent on the specific antibody as well as the distribution of the target protein and the specific behavior of the target cell within selected organs. Using spinning-disk confocal microscopy with 3D reconstruction capabilities revealed that immobilized Kupffer cells via FcγRII and FcγRIII grabbed crawling invariant Natural Killer T (iNKT) cells in the presence of an antibody (CXCR3-173). However, instead of inducing phagocytosis or any other form of cell death, Kupffer cells repeatedly ripped off the trailing edge of these crawling cells which ultimately led to depletion of iNKT cells in the liver. This is strikingly different from phagocytosis and we term this antibody-dependent fragmentation. We also show that this can be an extremely selective and efficient approach by demonstrating that depletion of these iNKT cells by antibody resulted in identical effects as a knockout mouse devoid of iNKT cells.

## Results

### iNKT cells in liver are specifically depleted by an anti-CXCR3 antibody (CXCR3-173)

**Figure 1:**
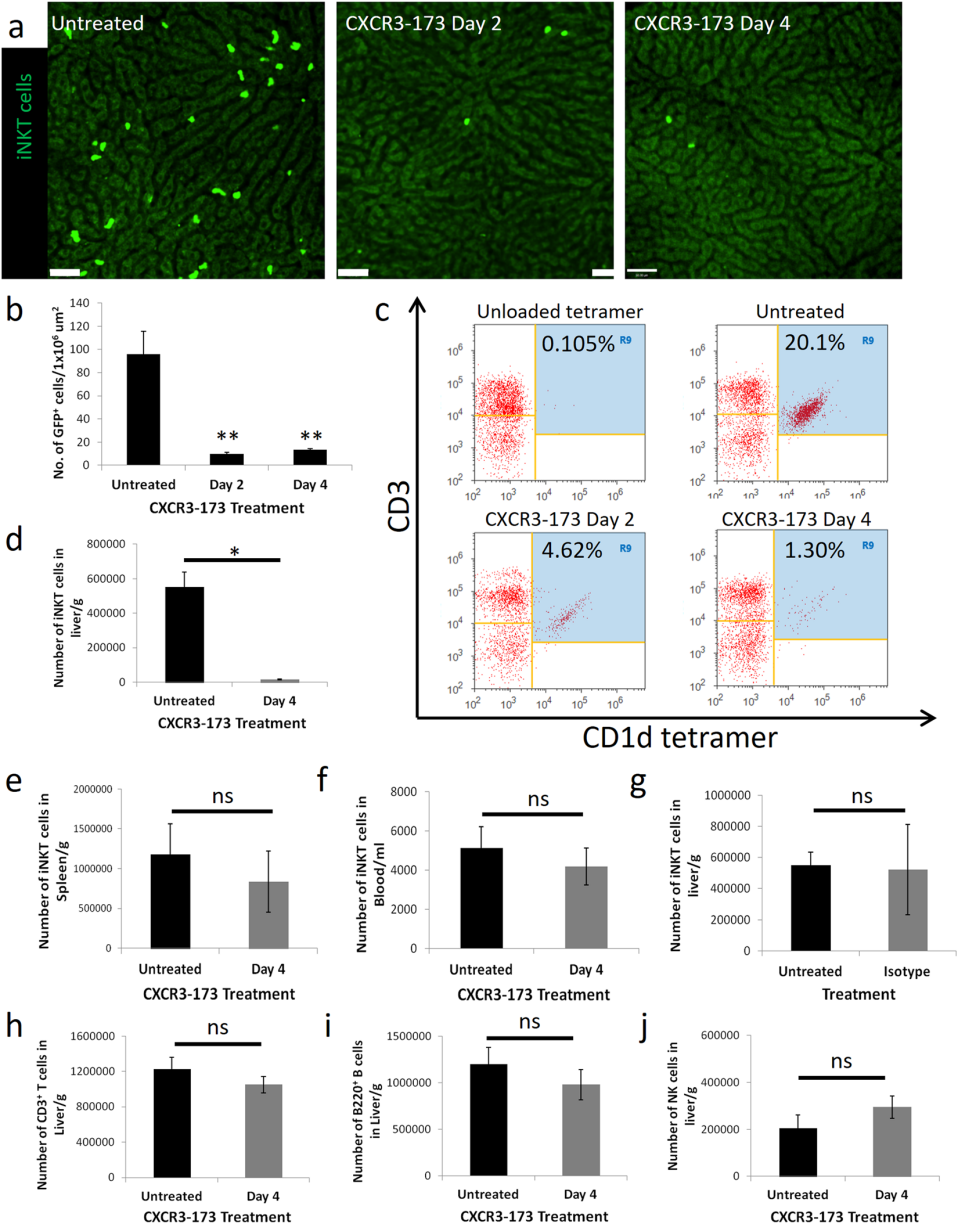
iNKT cells in the liver are depleted by CXCR3-173 antibody. (**a**) Representative intravital images of CXCR6-GFP liver 0, 2 and 4 days after CXCR3-173 antibody treatment from 3 mice. Bright green: iNKT cells. Scale bar: 50 μm. (**b**) Enumeration of GFP^+^ cells from intravital microscopy after treatment with CXCR3-173 antibody, n = 3 for all mice. **P < 0.01 by one-way ANOVA. (**c**) Representative flow cytometric plot demonstrating decrease of iNKT cells in liver after CXCR3-173 antibody treatment over 4 days, see panel (d) below for number of mice used. (**d**) Absolute number of iNKT cells in the liver after CXCR3-173 treatment at day 4, untreated: 7 mice, CXCR3-173 day 4: 4 mice, *P < 0.05 by *t* test. (**e**) Absolute number of iNKT cells in spleen after CXCR3-173 antibody treatment, untreated: 8 mice, CXCR3-173 day 4: 4 mice. (**f**) Absolute number of iNKT cells in peripheral blood after CXCR3-173 antibody treatment, untreated: 6 mice, CXCR3-173 day 4: 7 mice. (**g**) Absolute number of iNKT cells in liver after isotype antibody treatment, untreated: 7 mice, isotype control: 4 mice. Absolute number of (**h**) CD3^+^ T cells, untreated: 7 mice, CXCR3-173 day 4: 4 mice (**i**) B220^+^ B cells, untreated: 7 mice, CXCR3-173 day 4: 4 mice (**j**) NK (NKp46^+^CD3^−^) cells in liver after CXCR3-173 antibody treatment, untreated: 3 mice, CXCR3-173 day 4: 5 mice. All error bars are SEM.

CXCR3-173 antibody was injected intravenously into CXCR6-GFP mice, in which at least 60–80% of GFP^+^ cells are iNKT cells in the liver^38–40^. The distribution of iNKT cells up to 4 days after antibody treatment was visualized and quantified using intravital microscopy. To obtain a comprehensive perspective on the exposed liver lobe, 70 different fields of view were captured sequentially and these images were stitched into a single view (Suppl Figure S1). In the liver of untreated mice, iNKT cells were distributed evenly throughout the liver and resided exclusively in the sinusoids (see Suppl Movie S1), as described previously^38–40^. Two days and four days after CXCR3-173 treatment, a 10-fold decrease was noted in the number of GFP^+^ cells in the liver (Fig. 1a,b and Suppl Figure S1).

To confirm that intravenous CXCR3-173 treatment caused a specific depletion of the iNKT cell population, the liver, lung, spleen, joints, intestine and blood lymphocytes were isolated for flow cytometric analysis. The data confirmed that hepatic iNKT cells (CD45^+^B220^−^CD3^+^CD1d tetramer^+^) were depleted after four days (Fig. 1c,d). There was no change in the iNKT cell population in the spleen or blood after CXCR3-173 treatment (Fig. 1e,f) or other tissues we examined (such as the joints and intestine). To exclude the possibility of non-specific iNKT cell depletion due to the simple injection of antibodies into blood, experiments were repeated with an isotype control antibody. iNKT cells in the liver were not depleted after injection of the isotype antibody (Fig. 1g). In addition, CXCR3-173 treatment also reduced iNKT cells in the lung after four days, another organ in which iNKT cells have been described to crawl in blood vessels. Herein, we focus on the effect of CXCR3-173 on iNKT cells in the liver.

Other cell types including T cells, B cells and NK cells have been described to express the CXCR3 receptor^41, 42^ although these cells do not patrol the sinusoids like iNKT cells. This antibody has been previously described not to deplete naïve and activated CD4^+^ T cells or CD44^+^ memory T cells even with administration of up to 1 mg of given antibody^43^. To further examine if other cells are depleted, the number of T cells, B cells and NK cells was examined at 2 days and 4 days after CXCR3-173 treatment using flow cytometry. No change was observed in whole number of CD3^+^ T cells, B220^+^ B cells or NK cells in the liver (Fig. 1h–j). It is important to state that while 60–80% of CXCR6-GFP cells appear to be iNKT cells in the liver (therefore, 20–40% of CXCR6-GFP cells that are non-iNKT cells should not be depleted), 90% of CXCR6-GFP cells were depleted as observed by intravital microscopy. Either we are depleting another cell type, i.e. innate lymphoid cells (ILCs are also known to be CXCR6^+^) or we are under-representing how many CXCR6-GFP cells are iNKT cells. The latter is a likely possibility because a number of iNKT cells could internalize their TCR during the process of isolating iNKT cells from the liver. As a result, it might appear that there are fewer CXCR6-GFP iNKT cells by flow cytometry.

### iNKT cell depletion in liver occurs through fragmentation by Kupffer cells

**Figure 2:**
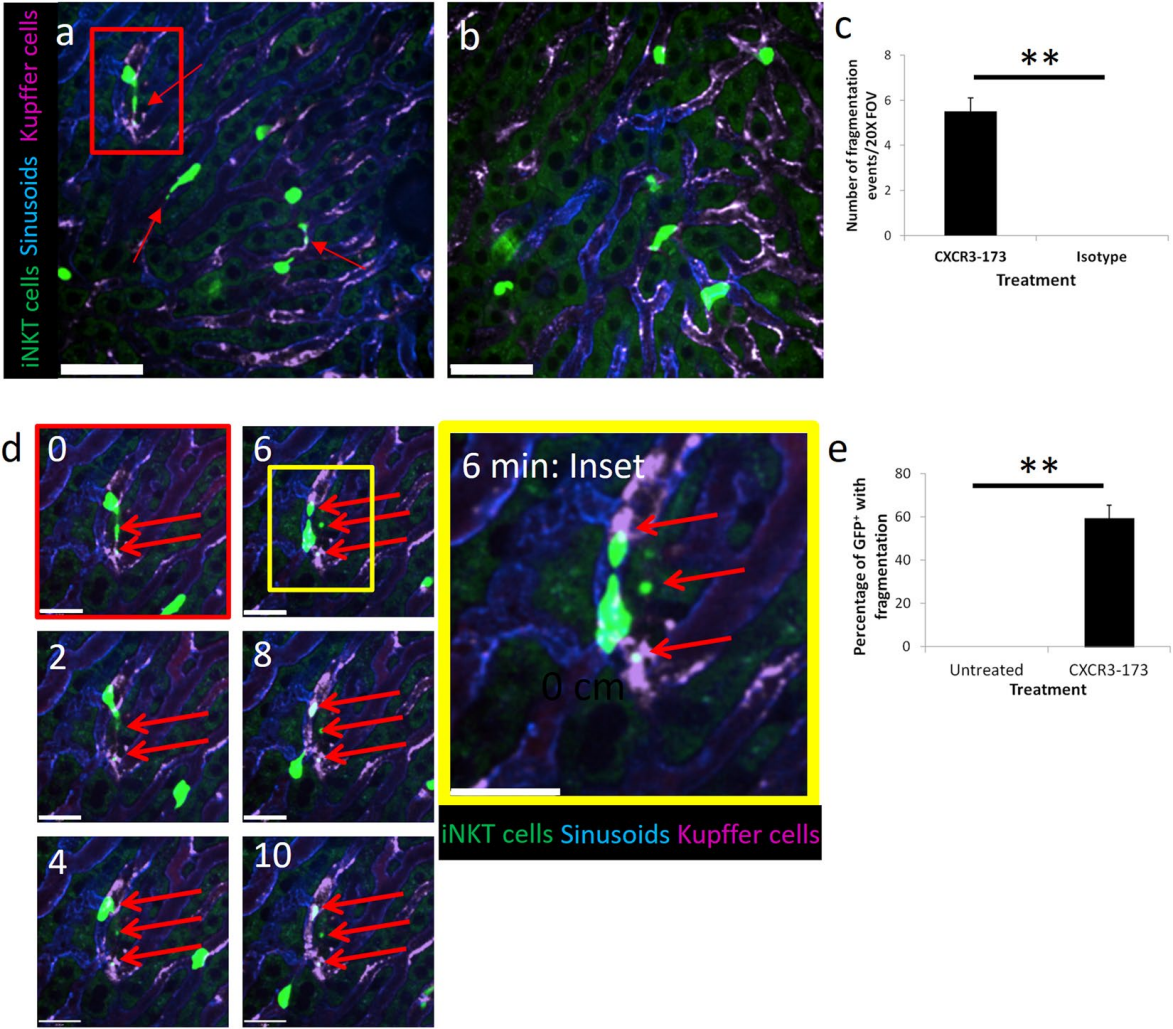
iNKT cell fragmentation by Kupffer cells. (**a**) Representative intravital images of iNKT cells fragmentation after CXCR3-173 treatment from 4 mice. Bright Green: iNKT cells, Blue: Alexa Fluor 647-labelled PECAM-1, Magenta: Alexa Fluor 750-labelled F4/80^+^ Kupffer cells. Scale bar: 50 μm. iNKT cell in red inset used for panel (**d**) demonstrating fragmentation over time. (**b**) No fragmentation of iNKT cells occurred with isotype control treatment from 3 mice. Bright Green: iNKT cells, Blue: Alexa Fluor 647-labelled PECAM-1, Magenta: Alexa Fluor 750-labelled F4/80^+^ Kupffer cells. Scale bar: 50 μm. (**c**) Quantification of fragmentation after CXCR3-173 or isotype control, CXCR3-173 treatment: 4 mice, isotype treatment: 3 mice, **P < 0.01 by *t* test, error bars are SEM. (**d**) Time-lapse intravital images from panel (a) demonstrating fragmentation of single iNKT cell; inset: enlarged image of fragment ripped from iNKT cells by Kupffer cells at 6 min post CXCR3-173 antibody treatment. Scale bars: 25 μm. (**e**) Percentage of GFP^+^ cells that undergo fragmentation after CXCR3-173, untreated: 5 mice, CXCR3-173 treatment: 4 mice, **P < 0.01 by *t* test, error bars are SEM.

**Figure 3:**
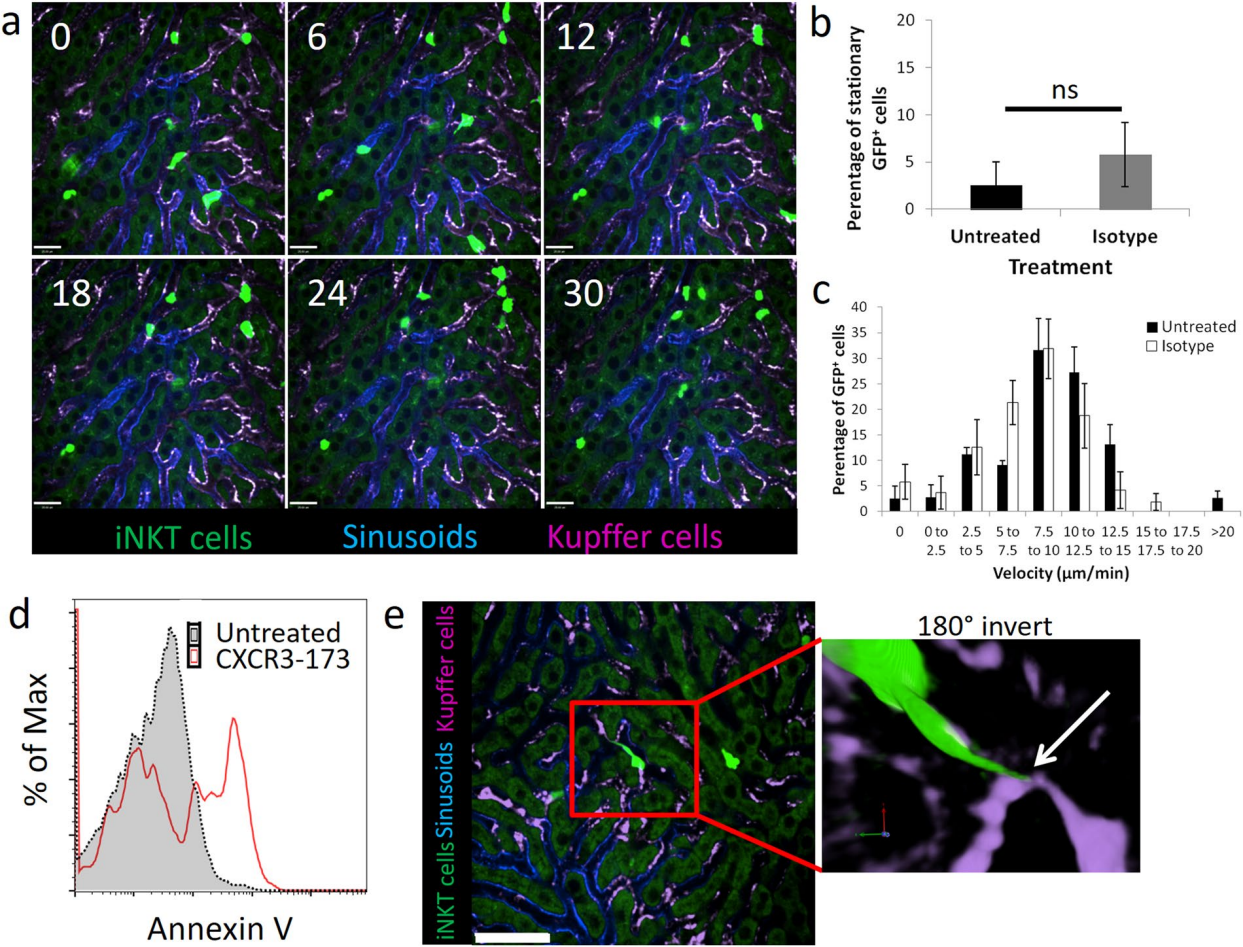
Isotype antibody did not alter iNKT cell behavior and iNKT cell-KC interaction after CXCR3-173 treatment leads to annexin V expression. (**a**) Representative intravital imaging of CXCR6-GFP mice immediately after treatment with isotype antibody from 3 mice. Bright green: iNKT cells, blue: Alexa Fluor 647-labelled PECAM-1, Magenta: Alexa Fluor 750-labelled F4/80^+^ Kupffer cells. Numbers in top left corner represents minutes of video recording. (**b**) Percentage of stationary iNKT cells from cell tracking after 30 mins of intravital imaging, untreated: 4 mice, isotype control: 4 mice. (**c**) Velocity frequency distribution of iNKT cells under basal and isotype antibody treated conditions, untreated: 4 mice, isotype control: 4 mice. All error bars are SEM. (**d**) Annexin V expression in CD45^+^CD3^+^CD1d-tetramer^+^ hepatic iNKT cells with (red line) and without (grey-scale) CXCR3-173 treatment. (**e**) Representative 3D reconstruction image of tail-end (white arrow) of crawling iNKT cell contacting KCs during fragmentation after CXCR3-173 antibody treatment from 4 mice. Green: iNKT cell, Magenta: Alexa Fluor 750-labelled F4/80^+^ Kupffer cells. Scale bars: 50 μm.

A previous study has demonstrated that the liver was a major site for B cell depletion after anti-CD20 treatment where Kupffer cells mediate the arrest and engulfment of B cells circulating in liver sinusoids^5^. To investigate if iNKT cell depletion after CXCR3-173 treatment is mediated by Kupffer cells, intravital imaging of the liver of CXCR6-GFP mice was performed. In the liver, iNKT cells crawled in a random pattern under basal conditions (Suppl Movie S1), at times changing directions, as previously described^39, 40^. Kupffer cells were sessile macrophages that reside in the lumen of sinusoids. iNKT cells crawled over Kupffer cells without a change in velocity or cell behavior (shown later). Strikingly, within minutes of CXCR3-173 antibody injection, iNKT cells began leaving GFP^+^-fragments behind as they crawled across Kupffer cells (Fig. 2a,d, inset and Suppl Movies S2 and S3). As the iNKT cell remained motile during this event, the acquisition of fragments occurred primarily from the trailing end or tail of the crawling iNKT cell. One or multiple fragments were removed from GFP^+^-iNKT cells as they traversed the immobilized Kupffer cell with or against blood flow (Fig. 2a,d and Suppl Movies S3 and S4). Approximately 60% of GFP^+^ cells had fragments acquired from the trailing end of the cell (Fig. 2e). Fragmentation continued to occur until the cell became so small that it stopped crawling. Isotype control antibody treatment caused no fragmentation (Fig. 2b,c) or alterations in iNKT cell behavior (Fig. 3a–c). As early as 24 hours after CXCR3-173 antibody treatment, a very noticeable number of iNKT cells were smaller from flow cytometric analysis (Suppl Figure S2). Other forms of cell death including phagocytosis and lysis were never observed.

Numerous forms of cell death including apoptosis, necrosis and autophagy have increased annexin V binding as cellular corpses ultimately lose their plasma membrane integrity^44–48^. Following fragmentation, annexin V expression was also detected in hepatic iNKT cells (Fig. 3d). We also performed Z-stack image acquisition and 3D reconstruction after CXCR3-173 antibody treatment. The trailing edge of the crawling iNKT cell appeared to be attached to the Kupffer cell, stretching the iNKT cell, prior to the tail becoming a fragment in the Z-stack 3D image (Fig. 3e and inset). Whole-cell phagocytosis of hepatic iNKT cells was not observed after CXCR3-173 treatment.

**Figure 4:**
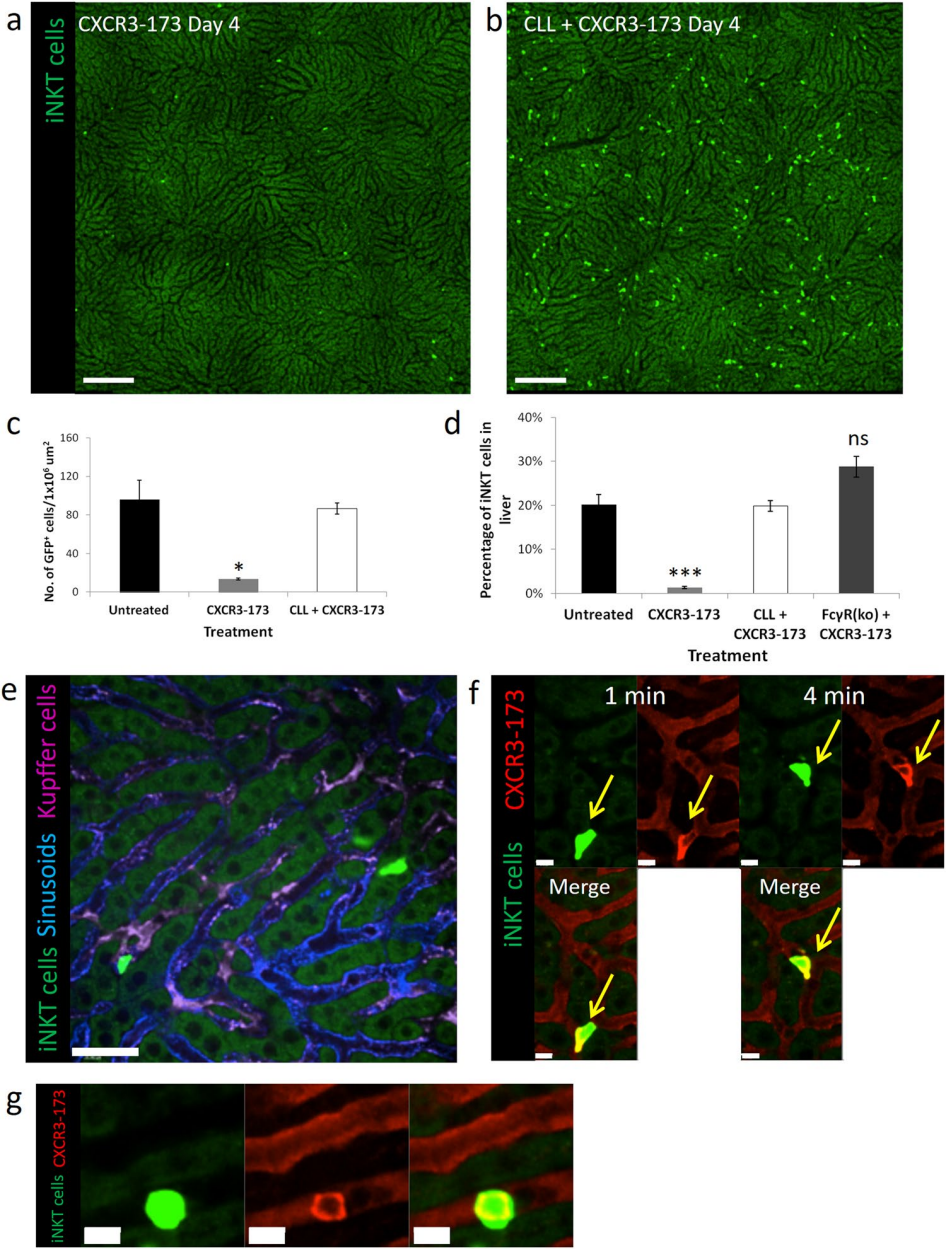
iNKT cell depletion in the liver is dependent on Kupffer cells and mediated by Fcγ receptor. (**a**,**b**) Representative intravital image of CXCR6-GFP mice treated with (**a**) CXCR3-173 antibody alone or (**b**) tandem CLL and CXCR3-173 antibodies. See panel (**c**) below for number of mice used. Bright green: iNKT cell. Scale bar: 200 μm. (**c**) Enumeration of GFP^+^ cells from intravital image after treatment with either CXCR3-173 antibody alone or CLL and CXCR3-173 antibodies, untreated: 4 mice, CXCR3-173: 3 mice, CLL + CXCR3-173: 3 mice, *P < 0.05 by one way ANOVA, error bars are SEM. (**d**) Percentage of iNKT cells in liver after CXCR3-173 antibody treatment alone or in tandem with CLL treatment. Livers are harvested at 4 days after treatment and percentage of iNKT cells were determined by flow cytometry (CD3^+^CD1d-tet^+^ cells), untreated: 7 mice, CXCR3-173: 4 mice, CLL + CXCR3-173: 3 mice, FcγR(KO) + CXCR3-173: 3 mice, ***P < 0.001 by one way ANOVA, error bars are SEM. (**e**) Representative ntravital image of CXCR6-GFP mice pre-treated with anti-CD16/anti-CD32 antibodies prior to CXCR3-173 antibody treatment from 3 mice. Bright Green: iNKT cells, Blue: Alexa Fluor 647-labelled PECAM-1, Magenta: Alexa Fluor 750-labelled F4/80^+^ Kupffer cells. Scale bar: 50 μm. (**f**) Representative intravital image of tail-localized distribution of CXCR3 molecules (red) on the surface of crawling iNKT cells (green) *in vivo* over time from 4 different fields of view. Numbers in top right corner represents minutes of video recording. Scale bars: 10 μm. (**g**) Representative image showing homogenous distribution of CXCR3 molecules (red) on surface of non-crawling iNKT cells (green) from 3 different fields of view. Scale bars: 10 μm.

The Kupffer cells appear to be the main mechanism mediating the depletion of hepatic iNKT cells. Clodronate liposomes (CLL) injected intravenously removed all Kupffer cells at 36 hours after CLL injection. Normal levels of iNKT cells were seen in the liver in mice subjected to tandem CLL and CXCR3-173 treatment as opposed to complete depletion by CXCR3-173 antibody alone (Fig. 4a–c). Kupffer cells clearly accounted for all of the depletion of GFP^+^-iNKT cells as Kupffer cell-depleted mice given CXCR3-173 antibody and mice receiving nothing had identical iNKT cell numbers (Fig. 4c). This observation was also confirmed using flow cytometry where normal levels of iNKT cells in the liver were noted when Kupffer cells were depleted prior to giving the CXCR3-173 antibody (Fig. 4d).

The role of Fcγ receptors were examined in the fragmentation of iNKT cells *in vivo*. iNKT cells in the liver were not depleted 4 days after CXCR3-173 antibody treatment in Fcγ receptor-deficient mice (Fig. 4d). FcγRII and FcγRIII were blocked with anti-CD16/anti-CD32 antibodies in CXCR6-GFP mice before the CXCR3-173 antibody was injected. iNKT cells crawled over Kupffer cells with no fragment formation in the sinusoids similar to basal untreated conditions (Fig. 4e and Suppl Movie S5).

Distribution of CXCR3 molecules on the surface of GFP^+^-iNKT cells were examined *in vivo* by injecting small amounts of PE-conjugated CXCR3-173 antibody. The majority of iNKT cells had CXCR3 molecules which were concentrated towards the tail end of the cell (Fig. 4f). This non-uniform distribution was only seen on polarized cells as iNKT cells with a round morphology had homogenous distribution of CXCR3 (Fig. 4g).

### Crawling of iNKT cells is critical for the CXCR3-173 induced fragmentation

**Figure 5:**
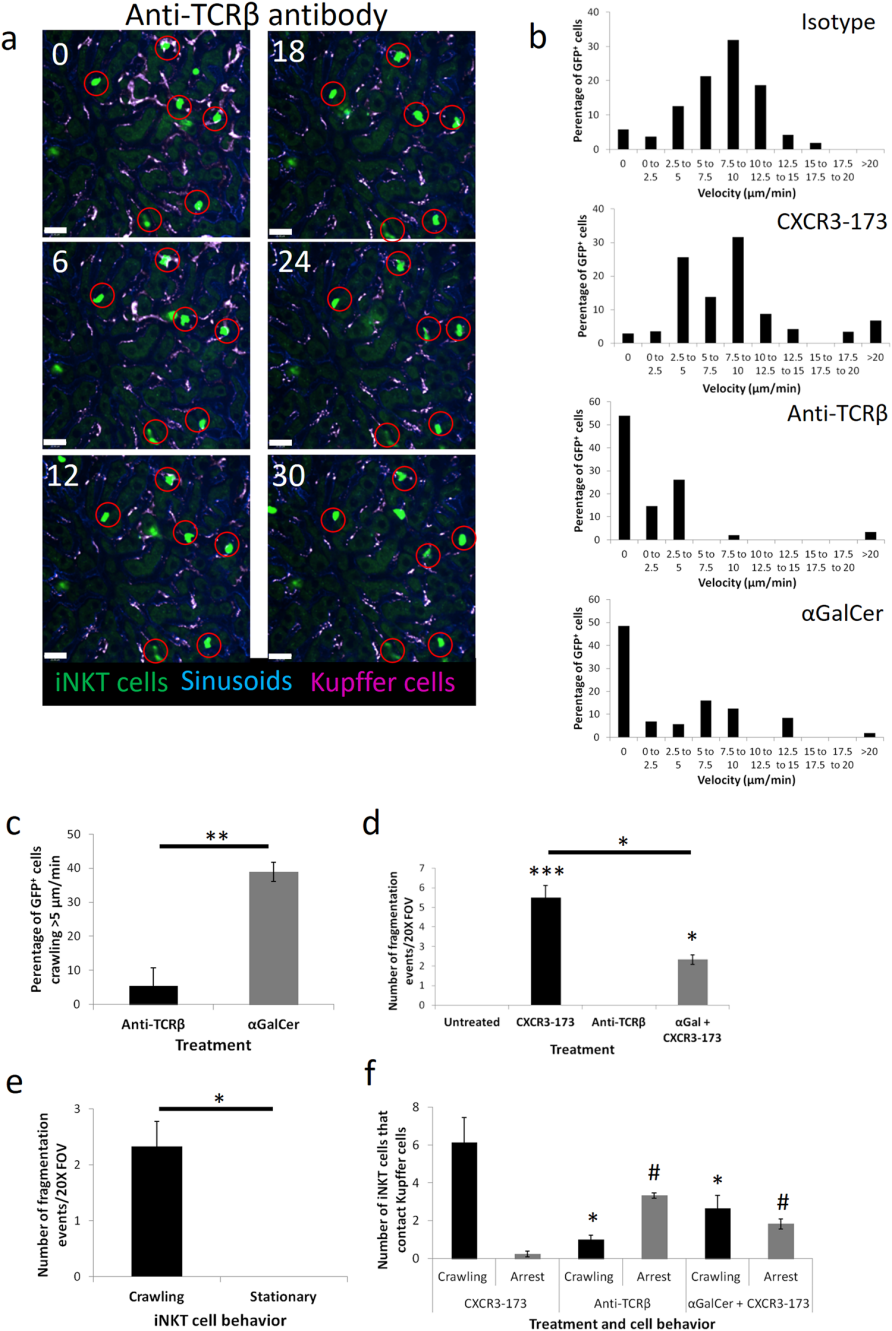
Fragmentation of iNKT cells is dependent on iNKT cell crawling. (**a**) Representative intravital image over time of GFP^+^ cells after anti-TCRβ antibody treatment from 3 mice. Scale bar: 50 μm. (**b**) Distribution of velocity frequencies of GFP^+^ cells in untreated mice (n = 4), anti-TCRβ (n = 3), CXCR3-173 (n = 3) or αGalCer (n = 3) treated mice. (**c**) Percentage of iNKT cells >5 μm/min after anti-TCRβ or αGalCer treatment, n = 3 mice, **P < 0.01 by *t* test, error bars are SEM. (**d**) Number of fragmentation events in field of view after antibody treatments, untreated: 5 mice, CXCR3-173: 4 mice, anti-TCRβ: 3 mice, αGalCer/CXCR3-173: 3 mice, *P < 0.05 by *t* test CXCR3-173 treatment against tandem aGalCer and CXCR3-173 treatment, ***P < 0.001 by one way ANOVA, error bars are SEM. (**e**) Number of fragmentation events in crawling or stationary iNKT cells after tandem αGalCer/CXCR3-173 treatment, n = 3 mice, *P < 0.05 by *t* test, error bars are SEM. (**f**) Kupffer cell contact by crawling or arrested iNKT cells with various treatments, CXCR3-173: 4 mice, anti-TCRβ: 3 mice, αGalCer + CXCR-173: 3 mice. *P < 0.05 by *t* test, CXCR3-173 crawling vs Anti-TCRβ crawling or CXCR3-173 crawling vs αGalCer + CXCR-173 crawling. ^#^P < 0.05 by *t* test, CXCR3-173 arrest vs Anti-TCRβ arrest or CXCR3-173 arrest vs αGalCer + CXCR-173 arrest.

The commonality between iNKT cells in liver and lung is that they crawl within blood vessels whereas this does not occur in spleen^49^ or joints^50^. Two approaches were used to stop iNKT cell crawling. First, anti-TCRβ antibody was given. Upon injection of anti-TCRβ antibody, 95% of iNKT cells slowed (<5 μm/min) or arrested (Fig. 5a,b and Suppl Movie S6). Under these conditions, essentially no fragmentation could be seen even if the arrested iNKT cell was on top of a Kupffer cell. This suggested that the fragments were being ripped from the crawling iNKT cell while fragmentation did not occur on stationary cells. Unexpectedly, when an iNKT cell stopped crawling on top of the Kupffer cell, the Kupffer cell did not phagocytose the iNKT cell suggesting that CXCR3-173 antibody exclusively induces fragmentation. In addition, α-galactosylceramide (αGalCer), a lipid ligand that results in the arrest of approximately 50% of iNKT cells, was injected^38, 51^. 40% of iNKT cells remained crawling at significant speeds (>5 μm/min) after αGalCer treatment (Fig. 5c). Tandem treatment of mice with αGalCer and CXCR3-173 decreased the number of cell fragmentation events by half (Fig. 5d). Intriguingly, only crawling iNKT cells were subject to fragmentation whereas arrested iNKT cells did not undergo fragmentation even if they were on top of a Kupffer cell (Fig. 5e and Suppl Movie S7). With fewer crawling iNKT cells, fewer of these cells contacted Kupffer cells after tandem αGalCer and CXCR3-173 treatment which accounted for the decrease in fragmentation events (Fig. 5d,f).

Since the iNKT cells were crawling after administration of the CXCR3-173 antibody, it seemed unlikely that the antibody was causing cell death via complement or ADCC. Nevertheless, Suppl Figure S3A demonstrated that addition of antibody to iNKT cells *in vivo* and then harvesting the cells for *in vitro* cell culture, revealed no direct untoward effects of this antibody to iNKT cells after 3 days. In addition, administration of the antibody to cultures of iNKT cells also caused no increase in apoptosis as determined by annexin V expression (Suppl Figure S3B and C).

**Figure 6:**
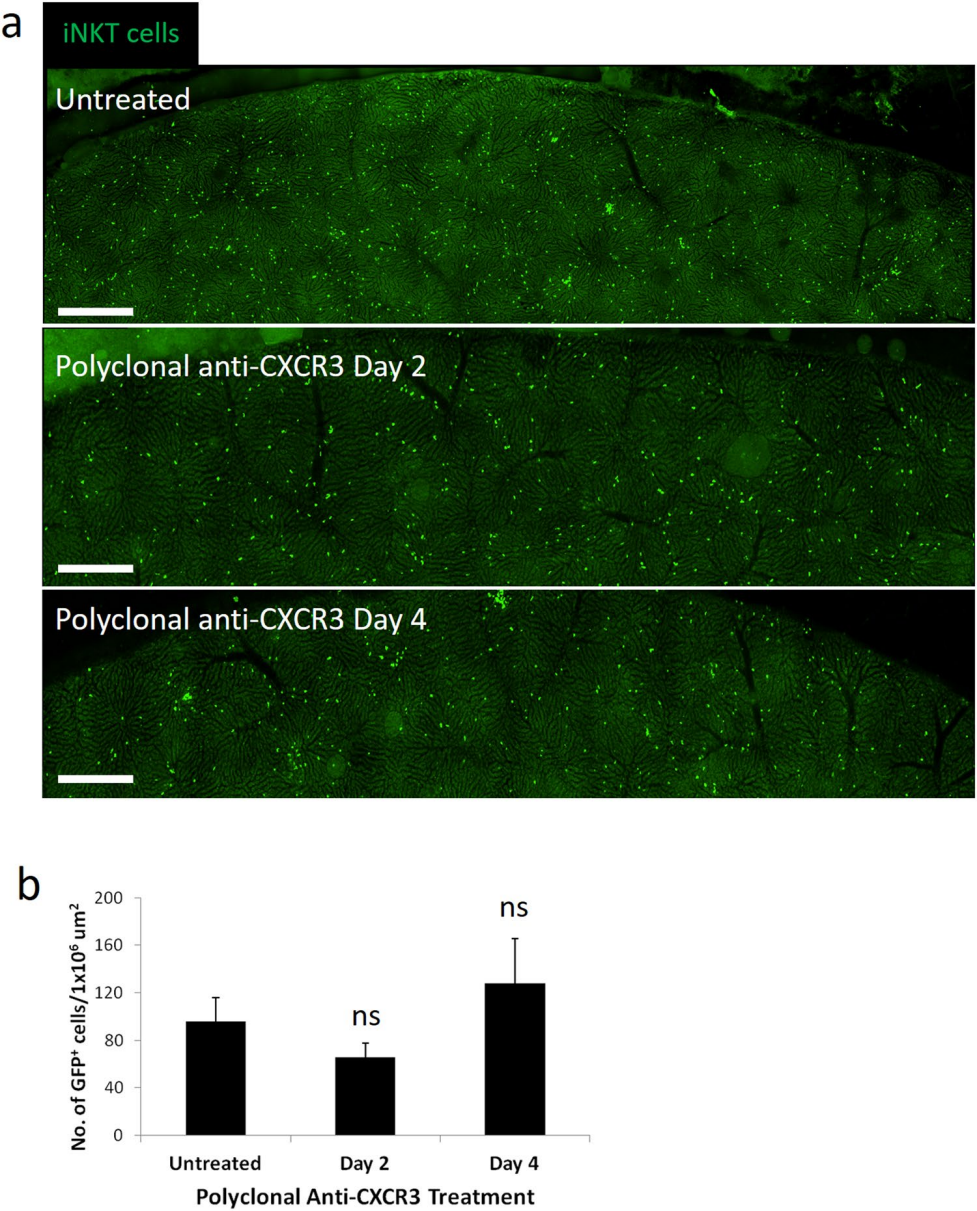
Polyclonal anti-CXCR3 antibody treatment does not deplete iNKT cells. (**a**) Representative stitched intravital image of the liver of CXCR6-GFP mice, 0, 2 and 4 days after polyclonal anti-CXCR3 antibody from 3 mice. Scale bars are 400 μm. (**b**) Quantification of number of GFP^+^ cells from stitched intravital image, n = 3 mice for all treatments. All error bars are SEM.

**Figure 7:**
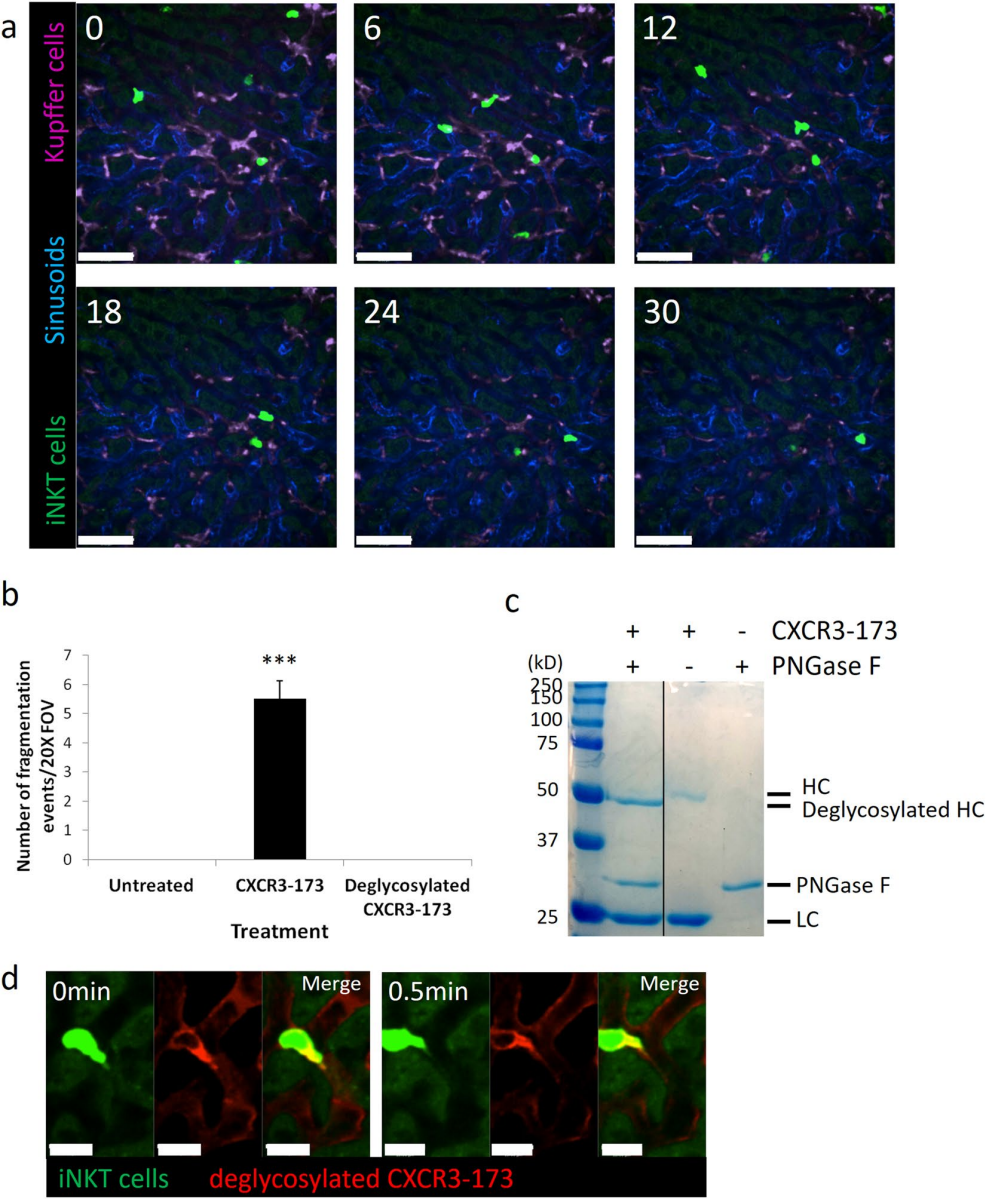
No fragmentation of iNKT cells with deglycosylated CXCR3-173 antibody. (**a**) Representative still images from intravital imaging demonstrating no iNKT cell fragmentation after *in vivo* injection of deglycosylated CXCR3-173 antibody from 3 mice. Bright Green: iNKT cells, Blue: Alexa Fluor 647-labelled PECAM-1, Magenta: Alexa Fluor 750-labelled F4/80^+^ Kupffer cells. Scale bar: 50 μm. (**b**) Enumeration of fragmentation events after antibody treatment, untreated: 5 mice, CXCR3-173: 4 mice, deglycosylated CXCR3-173: 3 mice, ***P < 0.001 by one way ANOVA, error bars are SEM. (**c**) SDS-PAGE analysis of untreated versus deglycosylated CXCR3-173 antibody. HC: heavy chain, LC: light chain. Gel image is cropped from SDS-PAGE analysis obtained from a single original gel. Full-length gel is presented in Supplementary Figure S3. (**d**) Representative intravital snapshots demonstrating binding of deglycosylated PE-conjugated CXCR3-173 antibody to iNKT cells from 3 different fields of view. Bright Green: iNKT cells, Red: deglycosylated PE-conjugated CXCR3-173 antibody.

To examine if other CXCR3 blocking antibodies could deplete iNKT cells, a rabbit polyclonal anti-CXCR3 antibody was injected into mice. Although this polyclonal antibody does not affect iNKT cell crawling under basal conditions, it was previously shown to block the chemotaxis of iNKT cells towards Kupffer cells (producing CXCL9) after Borrelia infection^50^. No significant decrease in iNKT cells were observed 2 days or 4 days after polyclonal anti-CXCR3 treatment suggesting that CXCR3-173 specifically causes fragmentation (Fig. 6a,b). All natural antibodies are glycosylated although glycan compositions and structures vary depending on host cells (i.e. mammalian origin). Alterations in glycoforms change binding affinities to Fc receptors (e.g. absence of glycosylation reduces or eliminates binding to Fc receptors) and result in modified immune effector functions^31, 52^. To induce fragmentation, a firm interaction between the Fc region of the antibody with the Fc receptor was predicted. To determine whether the glycosylation properties of the CXCR3-173 antibody induced the firm binding, CXCR3-173 antibody was deglycosylated with PNGase F. No fragmentation of iNKT cells was observed when deglycosylated CXCR3-173 was injected *in vivo* (Fig. 7a,b and Suppl Movie S8). Deglycosylation of CXCR3-173 antibody was confirmed by SDS-PAGE analysis where the heavy chain of the deglycosylated antibody migrated faster than the untreated control (Fig. 7c) which is consistent with previous observations^53, 54^. To confirm that the deglycosylated CXCR3-173 antibody could still bind to iNKT cells, PE-conjugated CXCR3-173 antibody was subjected to the same degylcosylation treatment before intravital imaging. Deglycosylated PE-CXCR3 was observed to localize to the trailing edge of the crawling iNKT cell (Fig. 7d) as previously observed (Fig. 4f). No degradation of the antibody was observed after deglycosylation treatment as visualized by SDS-PAGE.

### Validating iNKT cell depletion using CXCR3-173 antibody in liver-specific acetaminophen (APAP) overdose model

**Figure 8:**
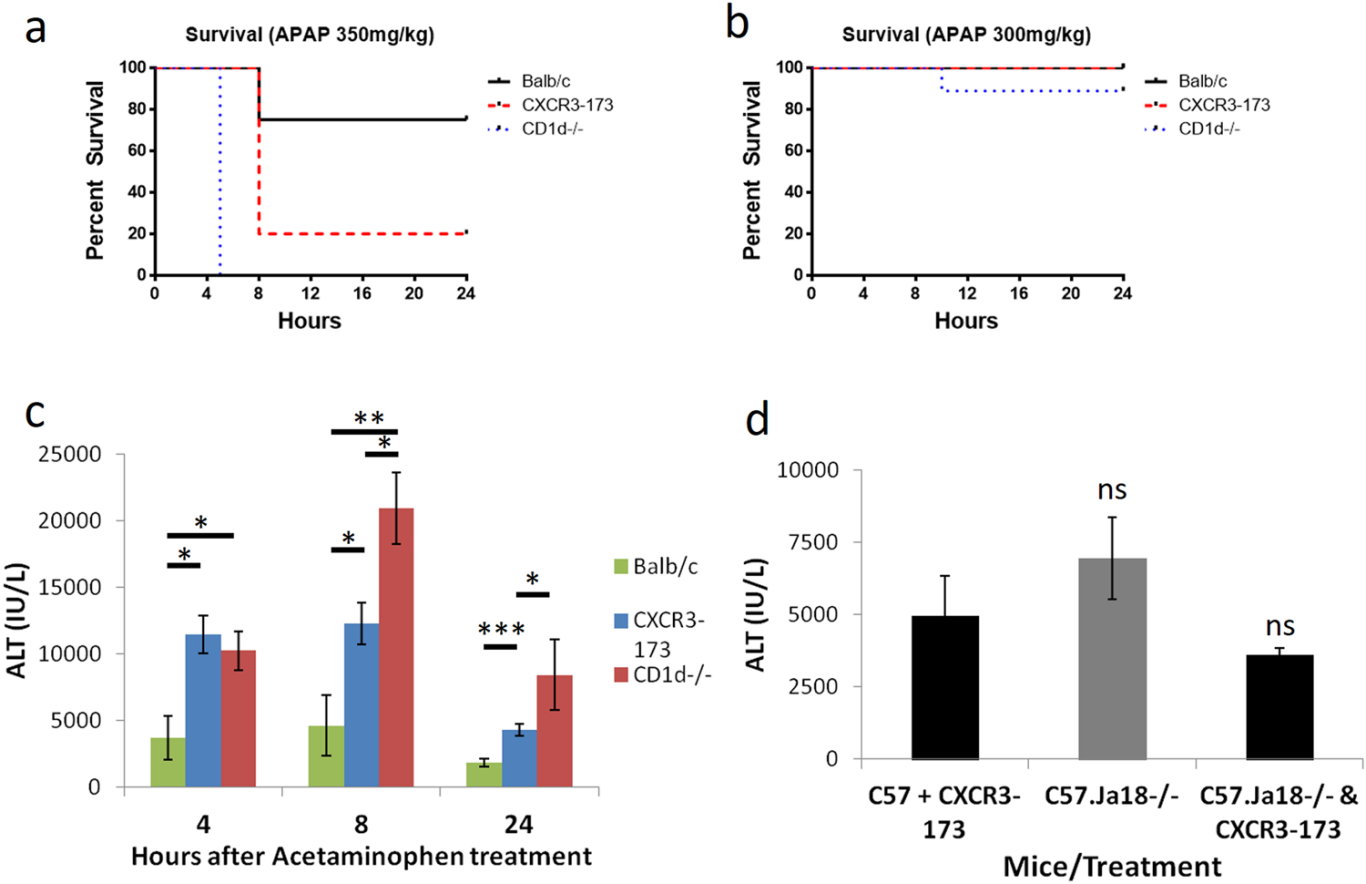
iNKT cells and acetaminophen overdose liver injury. (**a**) Survival of BALB/c (4 mice), CXCR3-173 treated mice (5 mice) or CD1d^−/−^ mice (5 mice) after 350 mg/kg acetaminophen (APAP) treatment. (**b**) Survival of BALB/c mice (8 mice), CXCR3-173 treated mice (4 mice) or CD1d^−/−^ mice (9 mice) after 300 mg/kg APAP treatment. (**c**) Serum ALT levels in BALB/c, CXCR3-173 treated mice or CD1d^−/−^ mice after 300 mg/kg APAP, 4 hours (BALB/c mice: 3 mice, CXCR3-173: 4 mice, CD1d^−/−^: 4 mice), 8 hours (BALB/c mice: 4 mice, CXCR3-173: 5 mice, CD1d^−/−^: 5 mice), 24 hours (BALB/c: 7 mice, CXCR3-173: 4 mice, CD1d^−/−^: 8 mice), *P < 0.05, *P < 0.01, *P < 0.001 by t-test, error bars are SEM. (**d**) Serum ALT in C57Bl/6 CXCR3-173 treated mice (4 mice), B6.Jα18^−/−^ mice (5 mice) or CXCR3-173 treated B6.Jα18^−/−^ mice (4 mice) 8 hours after 300 mg/kg APAP.

To ensure that the depletion of iNKT cells translated to impaired function, we used an APAP model of liver injury. APAP overdose results in severe, fulminant liver injury. Animal models of APAP-induced liver failure closely mirror those in human patients with formation of reactive metabolites, hepatocyte cell death and activation/contribution of immune cells to liver injury^55^. The role of iNKT cells in APAP overdose have been examined with genetic NKT cell knockout models, which suggests a protective role of iNKT cells^56^. At a dose of 350 mg/kg APAP, only 25% of wild-type mice died whereas all CD1d^−/−^ mice died (Fig. 8a), which was consistent with previously published data^56^. Similarly, 80% of mice treated with the CXCR3-173 antibodies did not survive. At a lower 300 mg/kg APAP dose, all mice survived till 24 hours with the exception of one CD1d^−/−^ mouse (Fig. 8b). This permitted the taking of blood samples. Marked increases in serum alanine aminotransferase (ALT) levels, as a marker of hepatocyte injury, were observed in all three groups of mice (Fig. 8c). However, the CXCR3-173 treated mice had significantly higher ALT levels than the wild-type mice and the CD1d^−/−^ mice had significantly higher levels than the CXCR3-173 antibody treated mice at 8 hours and 24 hours. From the ALT data, it was clear that the CD1d^−/−^ mice which lacked both type 1 iNKT and type 2 NKT cells, had greater injury than CXCR3-173 antibody treated mice in response to APAP-induced liver injury. As such, B6.Jα18^−/−^ mice, which lack only iNKT cells, were also subjected to APAP-induced liver injury and similar levels of serum ALT levels were detected in CXCR3-173 treated C57Bl/6 mice versus B6.Jα18^−/−^ mice (Fig. 8d). Further, B6.Jα18^−/−^ mice treated with CXCR3-173 antibody also had similar levels of serum ALT levels suggesting that CXCR3-173 antibody depleted only iNKT cells and not type 2 NKT cells (Fig. 8d). Treatment of wildtype mice with polyclonal anti-CXCR3 antibody, which does not deplete iNKT cells, did not increase serum ALT levels after acetaminophen treatment.

## Discussion

In our study, *in vivo* high resolution imaging using spinning disk microscopy revealed a pathway of cell death that required an antibody with functionally high binding to Fc, predominantly on Kupffer cells of the liver sinusoids. The latter is important as the process occurred under shear conditions and required motility by the target cell. The sessile Kupffer cell binds to the antibody that opsonized the crawling target cell which caused large fragments to detach from the crawling cell. Fragments were a few microns in diameter but sometimes were almost as large as the remaining cell. It is becoming clear that immune cells are able to “gnaw” at the plasma membrane of other cells and exchange proteins and/or remove proteins from a cell and internalize them. This phenomenon has been described as immune cell shaving or trogocytosis and can remove up to 80% of a molecule from the surface of a cell^57^. Due to the size of the particles, iNKT cell ripping by Kupffer cells did not appear to be a trogocytic type process. Rather, we coin the term antibody-dependent fragmentation to indicate that these were large fragments of the cell that were actively ripped from the target cell. It was not unusual to see a cell crawl across a Kupffer cell and leave behind a large fragment, reverse direction and traverse the Kupffer cell a second time leaving behind a second large fragment and this second fragmentation appeared to deliver a death blow; the iNKT cells stopped crawling and formed structures reminiscent of shistocytes or fragmented red blood cells.

There have been numerous mechanisms by which cell death can be induced including the autonomous cell death pathways: apoptosis and autophagy which triggers normal clearance of dying cells and prevents immune recognition of dead cell antigens. In contrast, cell death by necrosis usually occurs due to destructive perturbations that lead to cell lysis. These can occur when an immune cell releases toxic factors including oxidants, proteases and pore forming molecules to induce cell death. Another mechanism in which one cell eliminates another cell is phagocytosis, wherein an immune cell engulfs another cell. A recent study described the engulfment of B cells and B cell lymphoma through a two-step process following anti-CD20 antibody administration^5^. Initially, rapid attachment of circulating B cells to Kupffer cells occurred, followed by immediate phagocytosis. In our study, hepatic iNKT cells were not circulating but rather crawling within the sinusoids so there was no need for an initial capture. However, there was also no evidence of phagocytosis after antibody treatment. In contrast, Kupffer cells grabbed crawling iNKT cells via Fcγ receptors and tore fragments off crawling iNKT cells upon CXCR3-173 antibody treatment. This was not dependent on directional shear as iNKT cells crawling within flow would lose a portion of membrane, reverse direction, crawl against flow and lose another piece of their membrane. It was however, dependent upon the crawling by iNKT cells because arrest of iNKT cells, even on top of Kupffer cells, eliminated fragmentation and iNKT cell disappearance. This was also dependent on strong binding of the antibody through the Fc region by Kupffer cells and the amount of glycosylation present on the antibody. Although two different modes of iNKT cell arrest were used, one cannot exclude the possibility that activation rather than arrest prevents membrane ripping. However, the membrane ripping only occurred in organs where the iNKT cells crawled inside the vasculature and not in organs where iNKT cells were localized extravascularly (e.g. joint)^50^.

Our data would suggest that we depleted functionally iNKT cells within the liver, as their functional role in protecting the liver from toxic molecules like acetaminophen was absent after CXCR3-173 administration. Since iNKT cells can play important central detrimental roles in immune responses to autoimmune disease and liver fibrosis, this approach could be used to therapeutically intervene in the process^58, 59^. Extending this work to immunotherapy, one could now strategically design antibodies by altering glycosylation in Fc regions or transferring the Fc region of CXCR3-173 to Fabs or single chain Fragment variables (scFvs) to induce fragmentation of tumor cells and patrolling monocytes which are known to benefit tumor growth at the expense of the host. In addition, this antibody depleting approach could be useful as an experimental tool. NKT cell deficient mice have provided great insights into NKT cell biology but have certain limitations. CD1d deficient mice are also deficient in other CD1d restricted cells such as type 2 NKT cells and this subset of NKT cells are now emerging as important players in health and disease^60^. Finally, the CXCR3-173 antibody could be useful as an initial screen prior to lengthy breeding protocols of iNKT cell deficient mice onto various genetic mouse backgrounds or used as another tool to validate genetic knockout models.

Collectively, these studies establish fragmentation, as a novel cell death pathway mediated by Kupffer cells leading to antibody-mediated target cell depletion. With the ability to image interactions of immune cells at improved resolution with spinning disk intravital microscopy, we have provided significant insight into this effector mechanism and demonstrate that it is quite distinct from the phagocytotic mechanisms previously identified. Furthermore, we have identified a novel use of an antibody that can effectively deplete iNKT cells in the liver and lung. This creates new experimental possibilities that can increase our understanding of the role of iNKT cells in disease and facilitate development of novel therapeutic strategies.

## Methods

### Mice

BALB/c, Fcγ knockout and C57Bl/6 mice were purchased from The Jackson Laboratory. CXCR6-GFP knock-in mice on the BALB/c background were a gift from Dan R. Littman (New York University School of Medicine, New York). B6.Jα18^−/−^ mice were a gift from Brent Johnston (Dalhousie University, Halifax, Nova Scotia, Canada). All mice were maintained in a specific pathogen-free, double-barrier unit at the Faculty of Medicine, University of Calgary. All protocols used in this manuscript were approved by the University of Calgary Animal Care Committee (protocol #AC12-0163) and in accordance with guidelines established by the Canadian Council for the Use of Laboratory Animals.

### Antibodies and treatments

Armenian Hamster IgG functional grade purified anti-CXCR3 (clone CXCR3-173), Armenian Hamster IgG functional grade purified anti-TCRβ (clone H57-597), Armenian Hamster IgG functional grade purified non-binding isotype control (clone eBio299Arm), fluorescein isothiocyanate (FITC)-conjugated anti-CD45r (clone RA3-6B2), eFluor® 660-conjugated anti-CD3 (clone 17A2), FITC-conjugated anti-mouse NKp46 (clone 29A1.4), (Peridinin chlorophyll (PerCP)-conjugated anti-CD45 (clone 2D1), phycoerythrin (PE)-conjugated anti-CXCR3 (clone CXCR3-173) and allophycocyanin (APC)-Annexin V kit was purchased from eBioscience (San Diego, CA). Alexa Fluor 750-conjugated anti-F4/80 (clone BM8) was obtained from AbLab (University of British Columbia, Vancouver, BC, Canada). PBS57-loaded PE-conjugated mouse CD1d tetramer, which specifically binds to iNKT cells, was obtained from NIH (National Institutes of Health). α-GalCer was purchased from Funakoshi co. ltd (Tokyo, Japan). To deplete iNKT cells in liver and lung, 200 μg of CXCR3-173 was intravenously administered via tail vein two or four days prior to flow cytometry analysis or intravital microscopy. To investigate the mechanism of iNKT cell depletion, different treatments were used. Either 200 μg of CXCR3-173 or anti-TCRβ was injected via the jugular vein before imaging. In similar depletion mechanism experiments, α-GalCer (dissolved in 0.5% Tween 20 and 0.9% NaCl, 5 μg/mouse) with or without CXCR3-173 antibodies were injected via the jugular vein. Annexin V expression of iNKT cells was examined 24 hours after 200 μg of CXCR3-173 was injected via the tail vein. To investigate the role of Fcγ receptors in iNKT cell depletion after CXCR3-173 antibody treatment, 200 μg of anti-CD16/anti-CD32 were injected into CXCR6-GFP mice via the jugular vein 30 minutes before intravital microscopy and allowed to circulate before injection of CXCR3-173 antibodies. To visualize the liver vasculature, anti-PECAM-1 (clone 390) was conjugated to Alexa Fluor 647 using a protein labeling kit according to manufacturer’s instructions (Invitrogen, Eugene, OR) and injected via the jugular vein before intravital imaging. Kupffer cell depletion was performed by injecting 200 μl of clodronate liposomes via tail vein 36 hours prior to CXCR3-173 treatment as previously described^39^.

### Spinning-disk confocal intravital microscopy (SDIVM)

CXCR6-GFP mice were used for the visualization of hepatic iNKT cells^39, 40^. Intravital microscopy was performed with an Olympus IX81 inverted microscope (Olympus, Center Valley, PA), equipped with an Olympus focus drive and motorized stage (Applied Scientific Instrumentation, Eugene, OR). Images were acquired with 10×/0.40 UPLANSAPO and 20×/0.45 LUCPLANFLN objective lenses. The microscope was linked with a confocal light path (WaveFx; Quorum Technologies, Guelph, ON, Canada) based on a modified Yokogawa CSU-10 head (Yokogawa Electric Corporation, Tokyo, Japan). Activity of iNKT cells in the liver vasculature was acquired with four laser-excitation wavelengths in rapid succession (491 nm, 561 nm, 642 nm and 730 nm; Cobolt, Vortran and Omicron) and captured with appropriate band-pass filters (Semrock and Chroma). Typical exposure times for excitation wavelengths were 0.2–0.5 s. A 512 × 512 back-thinned electron-multiplying charge-coupled device camera (C9100-13, Hamamatsu, Bridgewater, NJ) was used for fluorescence detection. Z stacks of xy planes (0.5 μm intervals) were recorded with the inverted spinning-disc confocal microscope using either ASI focus drive (Applied Scientific Instrumentation) or Olympus focus drive (Olympus). Volocity software (Perkin Elmer, Waltham, MA) was used for 3D rendering, acquisition and analysis of images.

### Preparation of mouse liver for SDIVM

Preparation of the murine liver for intravital microscopy was performed as previously described^39^. Briefly, the jugular vein of an anesthetized mouse was cannulated to permit intravenous delivery of antibodies and additional anesthetic, as required. To maintain body temperature, mice were placed on a heating plate (CU-201, Live Cell Instruments) at 37 °C. A midline and lateral incision along the costal margin to the midaxillary line was performed to expose the liver. The mouse was placed on a right lateral position on the heating plate and the ligaments connecting the liver to the diaphragm were severed to allow externalization of the liver onto a glass coverslip. To prevent dehydration, exposed abdominal tissues were covered with saline-soaked gauze. A saline soaked KimWipe® disposable wipe was gently placed over the liver to restrict movement of the tissue on the slide and to prevent tissue dehydration.

### Liver, spleen, lung and blood cells isolation and flow cytometry analysis

Liver-, spleen-, and lung-derived lymphocytes were isolated from BALB/c mice using a method previously described^39^. Briefly, blood was collected from anesthetized mice by cardiac puncture and red blood cells were lysed with ACK lysis buffer (Lonza, Switerland). Leukocytes were first washed with cold PBS and resuspended in cold FACS wash buffer (FWB; PBS, 2% fetal calf serum, 0.5 mM EDTA). Livers and lungs were excised and finely minced in a digestive medium containing 0.05% collagenase type IV (Worthington Biomedical) and 0.002% DNase I in HBSS (for liver) or collagenase type I (Worthington Biomedical) and 0.002% DNase I in PBS (for lung). Concentrates were placed at 37 °C for 30 min with gentle agitation (for liver) or without (for lung). The spleen was excised and collected in cold PBS. Subsequently, single cell suspensions were generated from the liver and lung concentrates and spleen by a mechanism of disruption through a 40 μm nylon mesh. Suspensions were washed with ice-cold PBS (pH 7.4) and centrifuged at 300 × *g* for 10 min. Liver mononuclear cells (MNCs) were also additionally purified through a 37%/70% (vol/vol) Percoll gradient. All suspensions were then resuspended in cold FWB and counted in 0.4% trypan blue using a hemocytometer. All samples were analyzed on an Attune acoustic focusing cytometer (Applied Biosystems). For iNKT cell analysis, only MNCs were gated in FSC versus SSC flow cytometric plots. The absolute number of MNCs was standardized by tissue weight (for liver, lung and spleen) or milliliters for blood.

### Cell culture of iNKT cells

For *in vitro* cell culture experiments, hepatic iNKT cells from CXCR6-GFP mice were harvested 45 minutes after administration of CXCR3-173 antibody and incubated with RPMI-1640 media in 250 ml cell culture flasks for 3 days at 37 °C and 5% CO_2_. For *in vitro* CXCR3-173 incubation treatments, hepatic iNKT cells from BALB/c mice were harvested and incubated as described above for 24 hours. iNKT cells were incubated with CXCR3-173 for 30 minutes before staining for annexin V expression. Medium was supplemented with 10% fetal bovine serum, 1% penicillin/streptomycin cocktail and 1% Glutamax^TM^. All cell culture media and supplements were purchased from Thermo Fisher Scientific (Burlington, ON).

### Deglycosylation of antibodies

CXCR3-173 and PE-conjugated CXCR3-173 were deglycosylated under non-denaturing conditions. 200 μg of CXCR3-173 antibody was incubated with 1, 875 units of glycerol-free PNGase F (New England Biolabs, Ipswich, MA) according to manufacturer’s protocol for 24 hours at 37 °C before *in vivo* injection. 2 μg of PE-conjugated CXCR3-173 was incubated with 500 units of glycerol-free PNGase F for 24 hours at 37 °C before imaging studies. For SDS-PAGE analysis, 5 μg of CXCR3-173 antibody was incubated with or without PNGase F as described above before gel analysis. SDS-PAGE gel was stained with 0.25% coomassie blue (Sigma-Aldrich, Oakville, Ontario, Canada) solution dissolved in a methanol/acetic acid mixture.

### Acetaminophen (APAP) overdose treatment

Treatments were performed as described^56^. Mice were allowed food and water *ad libitum* until the start of experiment. Before treatment, mice were fasted overnight (16 hours). APAP was dissolved in warm saline and administered by intraperitoneal injection (350 mg/kg or 300 mg/kg doses). Food was restored immediately after APAP treatment. These doses are known to be sublethal in wild-type mice. After various time points, blood was harvested by cardiac puncture and liver tissues collected for histological analysis.

### Statistical analysis

For intravital imaging, a minimum of 3 mice (or more) per treatment group was used to generate an average value. In each independent mouse, two fields of view were used. All values are expressed in mean ± SEM. Data were compared with either unpaired Student’s *t*-test, one-way ANOVA with Bonferroni multiple comparisons post hoc test. Statistical significance was accepted at *P* < 0.05.

## Electronic supplementary material

Suppl Movie S1

Suppl Movie S2

Suppl Movie S3

Suppl Movie S4

Suppl Movie S5

Suppl Movie S6

Suppl Movie S7

Suppl Movie S8

Supplementary Figures and legends
