# Supplementary material for: Antibody-dependent fragmentation is a newly identified mechanism of cell killing in vivo
Source: Sci Rep. 2017 Sep 5;7:10515. doi: 10.1038/s41598-017-10420-z (PMC5585239; doi:10.1038/s41598-017-10420-z)
Supplement: Supplementary file 9 — Supplementary Figures and legends [file 41598_2017_10420_MOESM9_ESM.pdf]

Antibody-dependent fragmentation is a newly identified mechanism of cell killing *in vivo*.

Pei Xiong Liew[1], Jung Hwan Kim[1], Woo-Yong Lee[1], Paul Kubes[1,*]

[1]: Snyder institute of Chronic Diseases, University of Calgary, Calgary, Canada
[*]: Lead Contact

Corresponding author, Lead Contact:
Paul Kubes
HRIC 4AA16, University of Calgary
3330, Hospital Drive NW
Calgary, Alberta, T2N4N1
Canada
Email: pkubes@ucalgary.ca
Phone: 403-220-2705
Fax: 403-270-7516

Running title: Antibody-dependent fragmentation and cell death

SUPPLEMENTAL INFORMATION

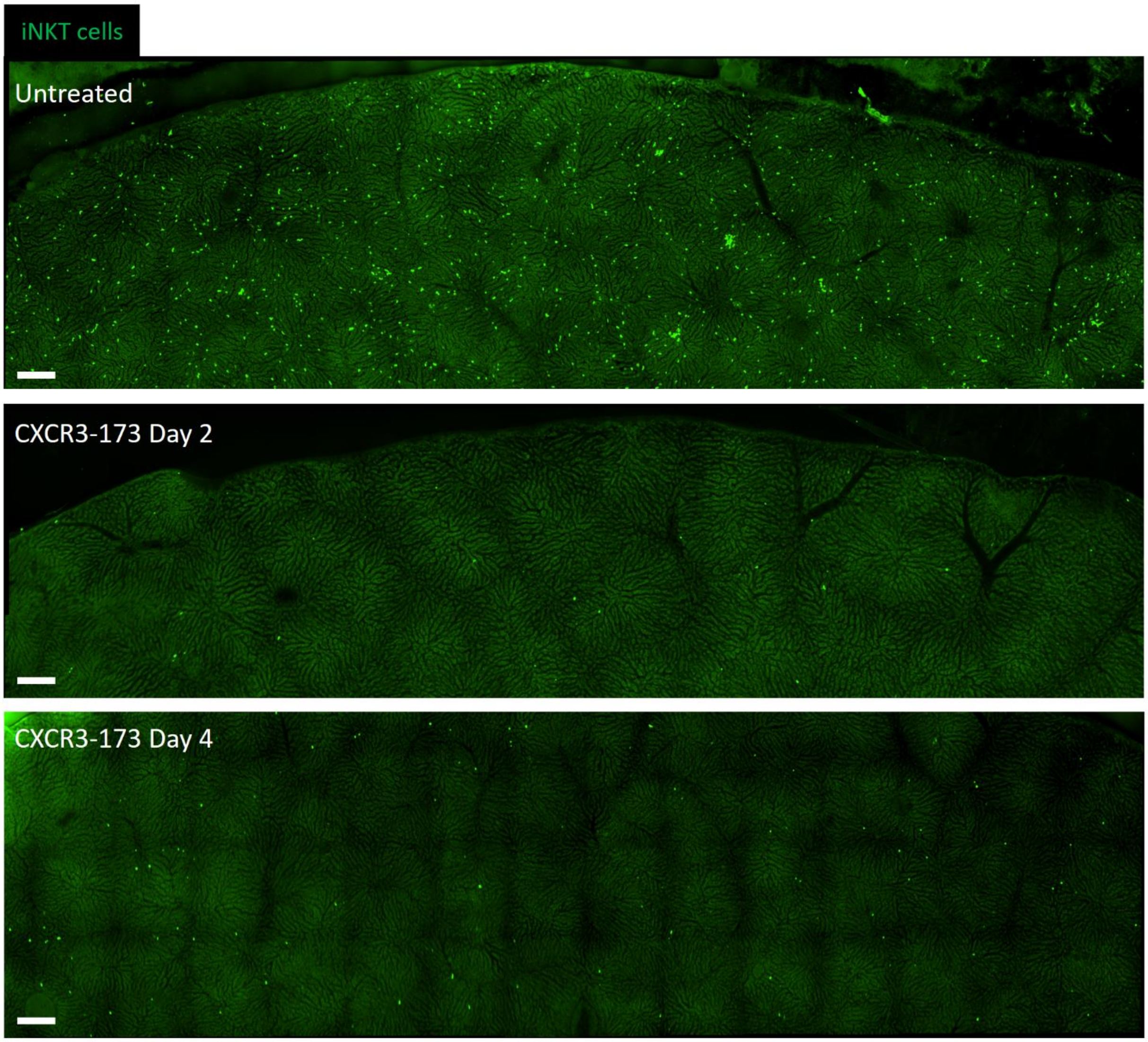

Suppl Figure S1: Stitched intravital image of CXCR6-GFP mice under untreated conditions, 2 or 4 days after CXCR3-173 antibody treatment. Scale bars are 400 µm.

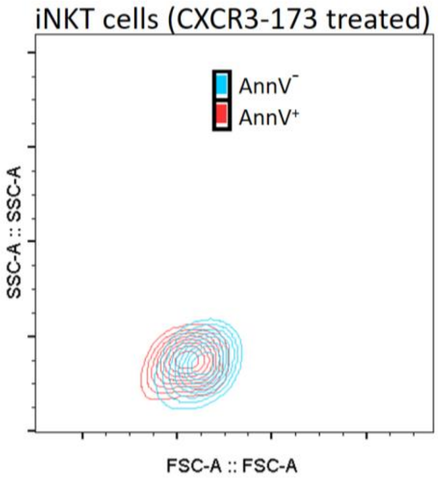

Suppl Figure S2: Flow cytometry forward and side scatter plot of hepatic iNKT "cells" that are Annexin V^+ (red) or Annexin V^- (blue) 24 hours after CXCR3-173 antibody treatment.

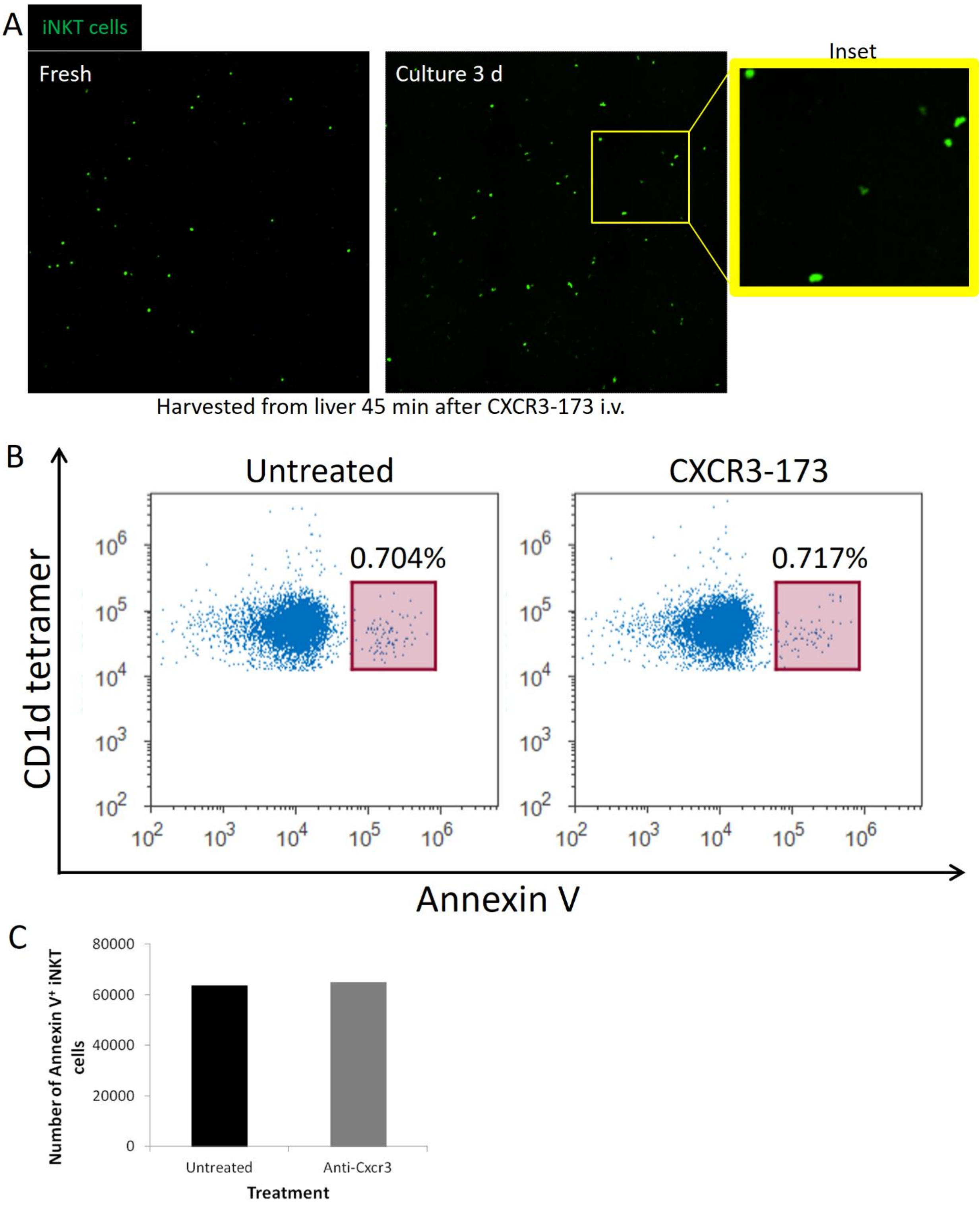

Suppl Figure S3: CXCR3-173 antibody alone is not lethal to iNKT cells. (a) 3 days culture of iNKT cells *in vitro* after injection of CXCR3-173 into mice. (b) Annexin V expression of iNKT cells after incubation with CXCR3-173 antibodies *in vitro*.

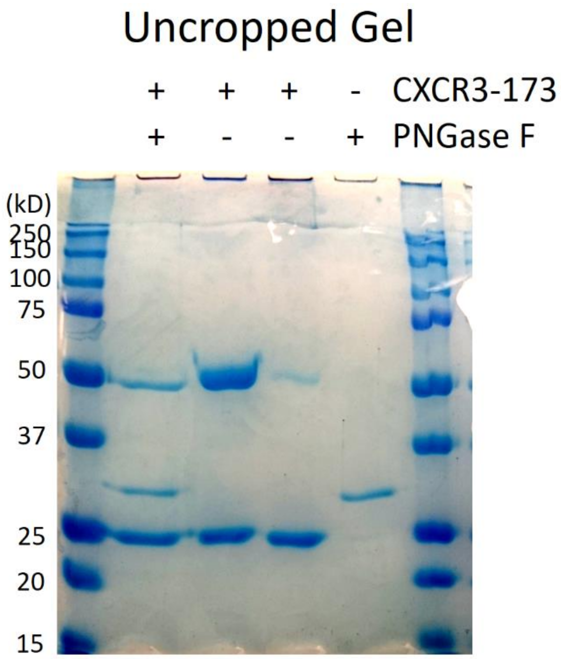

Suppl Figure S4: Uncropped SDS-PAGE gel from which Figure 7c is derived from. From left (lanes): Molecular weight marker, 5 µg CXCR3-173 treated with PNGase F, 25 µg CXCR3-173 without PNGase F, 5 µg CXCR3-173 without PNGase F, PNGase F alone, molecular weight marker.

SUPPLEMENTAL LEGENDS

Suppl Movie S1: **Localization and crawling behavior of hepatic iNKT cells under basal conditions**

iNKT cells (bright green) crawl randomly within liver sinusoids (blue) under normal conditions. Hepatocytes (dull green) were also observed to line the liver sinusoids. Intravital images were acquired using time-lapse confocal microscopy with a spinning-disc confocal microscope (Olympus IX81, Olympus). 4 frames were taken per minute for 30 minutes.

Suppl Movie S2: **Single fragment removed from one hepatic iNKT cell**

iNKT cell (bright green) loses a single fragment after CXCR3-173 treatment to Kupffer cells (magenta) in liver sinusoids (blue) after antibody treatment**.** Intravital images were acquired immediately after injection of antibody using time-lapse confocal microscopy with a spinning-disc confocal microscope (Olympus IX81, Olympus). 4 frames were taken per minute for 30 minutes.

Suppl Movie S3: **Fragmentation of hepatic iNKT cells after i.v. injection of CXCR3-173 antibody**

iNKT cells (bright green) lose fragments as they crawl over Kupffer cells (magenta) in liver sinusoids (blue) with 200 µg of CXCR3-173 treatment. Intravital images were acquired immediately after injection of antibody using time-lapse confocal microscopy with a spinning-disc confocal microscope (Olympus IX81, Olympus). 4 frames were taken per minute for 30 minutes. Still images from this video were used in Figure 3.

Suppl Movie S4: **Multiple fragments from one hepatic iNKT cell were ripped by Kupffer cell**

iNKT cell (bright green) can lose multiple after CXCR3-173 treatment to Kupffer cells (magenta) in liver sinusoids (blue) after antibody treatment. Intravital images were acquired immediately after injection of antibody using time-lapse confocal microscopy with a spinning-disc confocal microscope (Olympus IX81, Olympus). 4 frames were taken per minute for 30 minutes. Still images from this video were used in Figure 3.

Suppl Movie S5: **No hepatic iNKT cell fragmentation observed with isotype antibody treatment**

iNKT cell (bright green) crawls over Kupffer cells (magenta) in liver sinusoids (blue) similar to basal conditions with isotype antibody control treatment. Intravital images were acquired immediately after injection of antibody using time-lapse confocal microscopy with a spinning-disc confocal microscope (Olympus IX81, Olympus). 4 frames were taken per minute for 30 minutes.

Suppl Movie S6**: Arrest of hepatic iNKT cells after anti-TCRβ treatment**

Majority of iNKT cells (bright green) arrest after injection of anti-TCRβ in liver sinusoids (blue). No fragmentation of phagocytosis by Kupffer cells (magenta) was observed. Intravital images were acquired immediately after injection of antibody using time-lapse confocal microscopy with a spinning-disc confocal microscope (Olympus IX81, Olympus). 4 frames were taken per minute for 30 minutes.

Suppl Movie S7: **Fragmentation of iNKT cells only occurred in crawling iNKT cells**

A portion of iNKT cells (bright green) arrested next to Kupffer cells (magenta) after αGalCer treatment in liver sinusoids (blue). Fragmentation of iNKT cells were only observed in crawling iNKT cells after CXCR3-173 treatment. Intravital images were acquired immediately after injection of antibody using time-lapse confocal microscopy with a spinning-disc confocal microscope (Olympus IX81, Olympus). 4 frames were taken per minute for 30 minutes.

Suppl Movie S8: **No fragmentation of iNKT cells with deglycosylated CXCR3-173 treatment**

No fragmentation of iNKT cells (bright green) were observed when 200 μg of CXCR3-173 was injected as iNKT cells crawled over Kupffer cells (magenta) in liver sinusoids (blue) similar to basal conditions. Intravital images were acquired immediately after injection of antibody using time-lapse confocal microscopy with a spinning-disc confocal microscope (Olympus IX81, Olympus). 4 frames were taken per minute for 30 minutes.
